# Varicella-Zoster Disease of the Central Nervous System in Immunocompetent Children: Case Series and a Scoping Review

**DOI:** 10.3390/vaccines12091086

**Published:** 2024-09-23

**Authors:** Dawid Lewandowski, Kacper Toczylowski, Malgorzata Kowalska, Milena Krasnodębska, Iryna Krupienko, Karolina Nartowicz, Magdalena Sulik, Artur Sulik

**Affiliations:** 1 Department of Pediatric Infectious Diseases, Medical University of Bialystok, Waszyngtona 17, 15-274 Bialystok, Poland; kacper.toczylowski@umb.edu.pl (K.T.); milena.krasnodebska@udsk.pl (M.K.); karolina.nartowicz@udsk.pl (K.N.); artur.sulik@umb.edu.pl (A.S.); 2 Department of Pediatric Surgery and Neurology, Medical University of Bialystok, Waszyngtona 17, 15-274 Bialystok, Poland; malgorzata.kowalska@udsk.pl; 3 Department of Pediatrics, Endocrinology, Diabetology with Cardiology Divisions, Medical University of Bialystok, Waszyngtona 17, 15-274 Bialystok, Poland; magdalena.sulik@udsk.pl

**Keywords:** meningitis, encephalitis, varicella, zoster, immunocompetent, VZV

## Abstract

Background: Varicella-Zoster Virus (VZV) is characterized by its ability to enter a dormant state within the body. When the wild or vaccine virus reactivates, it can lead to herpes zoster (HZ), which infrequently manifests as a neuroinfection. Objectives: The aim of the study was to analyze the clinical manifestations and outcomes associated with VZV reactivation in the CNS in immunocompetent children. Methods: We searched medical databases for case reports using the keywords “zoster”, “meningitis”, “encephalitis”, and “immunocompetent”. The inclusion criteria were age below 18 years, any gender, race, and ethnicity, no features or history of immunodeficiency, and confirmation of VZV reactivation through the detection of VZV DNA in the CSF. Patients were categorized into two groups: children experiencing the reactivation of the wild virus and children with the vaccine strain virus. Results: The cohort included six children hospitalized in our hospital and 49 children reported in the literature. In 37 (67%), a wild-type virus was detected, while in 18 (33%), an infection was caused by the vaccine strain. There were no differences in the clinical presentation between the two groups. A typical rash was observed in 32 (58%) children. Approximately 41 of the 55 children (75%) received antiviral treatment. Four patients experienced complications. Conclusions: Neither a history of VZV immunization nor the absence of a skin rash can definitively exclude VZV meningitis. It is important to note that any seemingly healthy child, regardless of recognized risk factors, could develop HZ meningitis.

## 1. Introduction

Varicella-Zoster Virus (VZV) is a member of the herpesviridae well-known for causing chickenpox (varicella). A distinctive hallmark of herpesviridae is viral latency, wherein VZV persists in sensory neurons in a latent state post-primary infection and subsequent VZV vaccination [1,2]. The reactivation of the virus produces a clinical syndrome called herpes zoster (HZ), or shingles. The disease’s characteristic symptoms manifest as dermatome-distributed skin lesions, originating from the resurgence of the virus from cranial nerve sensory ganglia or spinal dorsal root ganglia, traversing axons to reach the epidermis. Meningitis or encephalitis represents a rare manifestation of herpes zoster, arising from the reactivated virus’s migration into the central nervous system (CNS). The majority of children exhibiting neurological manifestations undergo complete recovery without enduring residual neurological disturbances [3].

The implementation of varicella vaccination programs marked a breakthrough development in VZV prophylaxis. The varicella vaccine obtained approval from the Food and Drug Administration in 1995. The estimated efficacy of a single-dose immunization is 85% and increases to 98% when two doses are implemented [4].

Following the introduction of the vaccine, the change in HZ morbidity was observed. Initial studies indicated an increase in HZ incidence compared to the pre-vaccine era, attributed mainly to the diminishing prevalence of natural varicella exposure and a subsequent reduction in wild virus circulation in society [5]. A study from 1965 postulated that natural VZV exposure acted as a preventive booster, maintaining latent VZV in check and thereby averting the onset of herpes zoster [6].

Contrary evidence has emerged in recent studies, revealing that vaccination not only serves as a deterrent against chickenpox, but also significantly reduces HZ incidence in vaccinated populations [7]. Children who received the VZV vaccine exhibited a 79% lower incidence of HZ compared to their non-vaccinated counterparts [8]. In Europe, a reduction exceeding 80% in disease occurrence and hospitalizations has been reported [9].

However, it is noteworthy that the virus present in the vaccine can undergo reactivation. Consequently, two distinct types of VZV causing HZ can be delineated: the wild-type and the vaccine strains, with the latter exhibiting lower virulence [10,11,12,13]. Furthermore, both the wild-type and vaccine strains may contribute to central nervous system (CNS) diseases, as elucidated in our study, which describes six cases of children infected with the wild-type and compares them with cases of VZV reactivation documented in the literature. To date, treatment standards for VZV reactivations in the CNS have not yet been developed. A scoping review was conducted in order to systematically map the research completed in this area, particularly regarding treatment and disease outcomes. Therefore, the aim of the study was to analyze clinical aspects of HZ reactivating as the infection of the CNS and to examine the differences between the two types of infection—vaccine-derived and wild-type.

## 2. Methods

### 2.1. Protocol

The protocol for this scoping review was based on the Preferred Reporting Items for Systematic Reviews and Meta-Analyses extension for Scoping Reviews (PRISMA-ScR) Checklist (available in Supplementary Materials File S1) [14].

### 2.2. Study Design

The aim of the study was to analyze the clinical manifestations and outcomes associated with VZV reactivation, manifesting as CNS infection, within the demographic of immunocompetent children aged less than 18 years. A comparative analysis was conducted between vaccine-associated infections and those arising from the wild-type virus. The study encompasses a retrospective analysis of a cohort comprising 55 patients, incorporating 49 cases extracted from existing literature and an additional 6 cases documented in the hospital records of the Department of Pediatric Infectious Diseases at the Medical University of Bialystok Children’s Clinical Hospital, Poland.

### 2.3. Search Strategy

**Figure 1:**
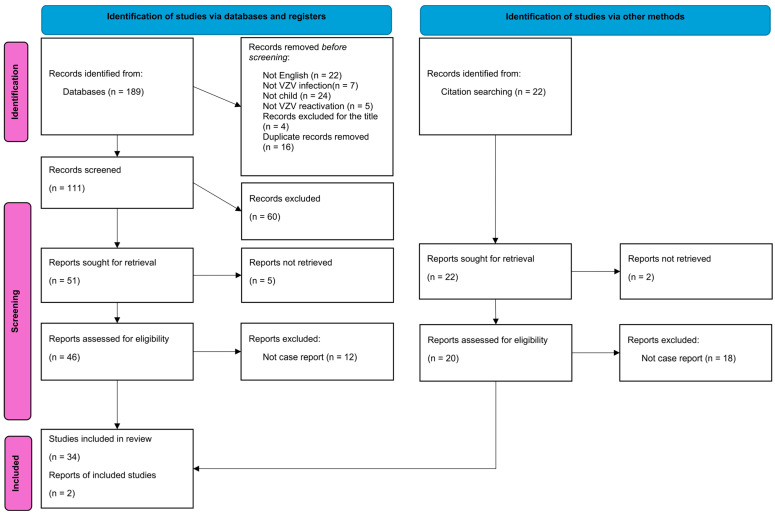
PRISMA flowchart illustrating the selection process of included studies. The Preferred Reporting Items for Systematic Reviews and Meta-Analyses (PRISMA) Flowchart depicts the step-by-step selection process of studies included in this systematic review. The flowchart outlines the number of records initially identified through database searching, the number of records screened and assessed for eligibility, and the final number of studies included in the review. Created with Microsoft Word, Microsoft 365 (http://www.microsoft.com/word).

We systematically searched PubMed and Google Scholar for all case reports and case series presenting clinical characteristics of patients with meningitis or encephalitis caused by the reactivation of a latent infection with Varicella zoster virus, using the keywords “zoster” and “meningitis” or “encephalitis” and “immunocompetent” (Figure 1). The search spanned from the inception of the database to 20 August 2022, yielding a total of 189 publications. The duplicate studies were removed, and three independent reviewers (D.L., K.T., and M.K.) performed a screening of the titles and abstracts and, in cases of disagreement, were included for full review. A data-charting form was collaboratively developed by three reviewers (D.L., K.T., and M.K.) to identify the variables to be extracted. All reviewers independently charted the data, reviewed the results together, and continuously refined the data-charting form through an iterative process.

### 2.4. Selection Criteria 

We included in the study all cases that fulfilled the following criteria: age below 18 years, any gender, race, and ethnicity, no features or history of immunodeficiency, confirmation of VZV reactivation through the detection of VZV DNA in cerebrospinal fluid using PCR reaction, and evidence obtained through interviews indicating either a history of primary VZV infection or prior VZV vaccination.

The exclusion criteria were as follows: studies published in languages other than English, original studies lacking a case report or case series presentation, those not addressing VZV reactivation, investigations involving patients older than 18 years, and/or those not meeting the criterion of immunocompetence.

The patient cohort was categorized into two groups: children experiencing the reactivation of the wild virus and those manifesting the vaccine strain reactivation. Children without a history of vaccination or who developed chickenpox despite immunization were classified under wild VZV reactivation. Conversely, those who received the VZV vaccine and remained free of chickenpox were categorized as presumed cases of vaccine strain reactivation. Confirmation of definite vaccine strain reactivation was achieved through genotype analysis of the VZV in the cerebrospinal fluid.

### 2.5. Data Collection 

Articles meeting the predefined inclusion criteria were submitted for full text screening. Two reviewers (D.L. and M.K.) independently charted the data from each eligible article. Any discrepancies were resolved through discussion between the two reviewers or by involving a third reviewer (K.T.) for further adjudication. Selection for review was based on the indication, either in titles or abstracts, that the articles reported individual patient or group data pertaining to immunocompetent subjects below the age of 18, with a diagnosis of meningitis or encephalitis caused by a reactivation of a latent Varicella-Zoster Virus infection. The data analysis focused on the patients’ characteristics, clinical manifestation, and treatment. The screening and selection process has been described in a PRISMA flowchart (Figure 1).

Additionally, we have reviewed the medical records of children hospitalized due to VZV CNS reactivation in the Medical University of Bialystok Children’s Clinical Hospital, Poland. We identified six cases meeting the predefined criteria outlined in the Selection Criteria section.

### 2.6. Statistical Analysis

Numerical variables with distributions deviating from the normal distribution were reported as medians with minimum and maximum values and compared using the Mann–Whitney U test. Categorical variables were reported as counts (n) and percentages (%) and compared using the chi-squared test. The significance level of the statistical tests in this analysis was set at α = 0.05. 

## 3. Results 

### 3.1. Patient Characteristics

A total of 49 immunocompetent children <18 years experiencing the reactivation of the VZV infection were identified in the literature. Additionally, we reported six more children hospitalized in our hospital in years 2017–2023 (Patient Nos. 50–55 in Table 1). In 37 (67%), a wild-type virus was detected, while in 18 (33%), an infection with a vaccine strain was diagnosed. The children presented with meningitis (n = 45; 82%), encephalitis (n = 3; 5.5%), meningoencephalitis (n = 4; 7%), and pseudotumor cerebri (n = 3; 5.5%).

Only three children with the infection caused by the wild-type virus were immunized with the varicella vaccine. In two of them, the vaccine was administered three-and-a-half [15] and five [16] years before the primary infection. In the third child, the vaccine was administered six years after the primary infection and five years before reactivation caused by the wild-type VZV [17]. Six (33%) children with VZV reactivation were immunized with two doses of the vaccine. Time intervals between primary exposure and reactivation were known for 41 patients and ranged from 1 to 14 years (median 10 years).

**Table 1:** Individual characteristics of patients with infection of the central nervous system caused by reactivation of varicella zoster virus. Abbreviations: M—meningitis; M/E—meningoencephalitis; E—encephalitis; P/C—pseudotumor cerebri.

Patient No.	Age (Y)	Sex	Wild/Vaccine Strain	Clinical Manifestations (Summary)	Rash (Dermatomal Distribution)	CSF Protein	CSF Glucose	CSF Wbc (%Lym)	Diagnosis	Antiviral Treatment	Source
1	5	Girl	Vaccine (presumed)	fever, headache, rash, meningeal signs	Trigeminal, Cervical	133	46	715 (97%)	M	Acyclovir I.V. (10 days)	[18]
2	8	Boy	Vaccine (confirmed)	skin rash, headache, photophobia, fever, meningeal signs	Cervical	36	68	94 (96%)	M	Acyclovir I.V. (9 days); Valacyclovir P.O. (7 days)	[19]
3	4	Boy	Vaccine (confirmed)	no detalis	Cervical	no info	no info	no info	M	Acyclovir (no details)	[20]
4	9	Boy	Vaccine (confirmed)	skin rash, left arm pain, headache, fatigue, pain in the neck, meningeal signs	Cervical	55	54	30 (79%)	M	Acyclovir I.V. (10 days)	[21]
5	3.5	Girl	Vaccine (confirmed)	skin rash, dizziness, vomiting, somnolence	Trigeminal	10	58	0	E	Acyclovir I.V. (15 days)	[22]
6	7	Boy	Vaccine (confirmed)	right arm pain, skin rash, fever, headache, photophobia, vomiting	Cervical	29	57	16 (71%)	M	Acyclovir I.V. (21 days)	[23]
7	12	Girl	Vaccine (confirmed)	rash, fever, elevated aminotransferases	Cervical	no info	no info	no info	M	no info	[24]
8	2	Boy	Vaccine (presumed)	fever, headache, periorbital edema, skin rash, fatigue, abdominal pain	Trigeminal	no info	no info	no info	M	Acyclovir I.V. (10 day)	[25]
9	7	Boy	Vaccine (presumed)	skin rash, fever, vomiting, meningeal signa	Trigeminal	no info	no info	no info	M	Acyclovir I.V. (8 days)	[26]
10	14	Girl	Vaccine (confirmed)	headache, skin rash, nausea, weakness, myalgias,	Thoracic	no info	no info	568 (92%)	M	Acyclovir I.V (7 days); Valacyclovir P.O. (14 days)	[27]
11	14	Boy	Vaccine (confirmed)	malaise, anorexia, skin rash, headache, photophobia, meningeal signs	Lumbar	64	60	140 (88%)	M	Acyclovir I.V. (7 days)	[28]
12	14	Girl	Vaccine (presumed)	headache, dizziness, nausea, vomiting, confusion, fever	Lumbar	132	45	775 (82%)	M	Acyclovir I.V. (7 days); Valacyclovir P.O (14 days)	[29]
13	7	Boy	Vaccine (confirmed)	headache, fever, vomiting, meningeal signs	No rash	60	50	544	M	Acyclovir I.V (4 days); valacyclovir P.O. (10 days)	[30]
14	12	Boy	Vaccine (confirmed)	fever, headache, phonophobia, photophobia, meningeal signs	No rash			83 (88%)	M	Acyclovir I.V. (5 days); acyclovir P.O (9 days)	[30]
15	12	Boy	Vaccine (confirmed)	skin rash, severe flank and thigh pain, headache, photophobia	Cervical, thoracic, lumbar	96		252	M	Acyclovir I.V. (10 days)	[31]
16	18	Boy	Vaccine (presumed)	headache, fever, nausea	No rash	44	59	234 (83%)	M	Acyclovir I.V. (12 days)	[32]
17	15	Girl	Vaccine (confirmed)	headache, fever, vomiting, meningeal signs	No rash	136	42	533 (88%)	M	Acyclovir I.V (14 days)	[33]
18	13	Boy	Vaccine (presumed)	headache, nausea, vomiting, fever, meningeal signs	No rash	136.7	45	609 (99.6%)	M	No treatment	[34]
19	3.5	Girl	Wild		Trigeminal	no info	no info	no info	M	no info	[35]
20	13	Girl	Wild		Trigeminal	no info	no info	no info	M	no info	[35]
21	15	Boy	Wild		Thoracic	no info	no info	no info	M	no info	[35]
22	2	Boy	Wild	fever, disturbed consciousness, vomiting, headache, rhinitis, somnolence	No rash	Normal	Normal	Normal	E	Acyclovir (15 days)	[36]
23	12	Boy	Wild	headache	No rash	86	47	590 (97%)	M	No treatment	[37]
24	8	Boy	Wild	skin rash, headache, low-grade fever, meningeal signs	Thoracic			47 (87%)	M	Acyclovir I.V. (7 days)	[38]
25	14	Boy	Wild	headache, vomiting, photophobia	No rash	158	35	285	M	Acyclovir (10 days); valacyclovir (4 days)	[39]
26	11	Boy	Wild	abdominal pain, vomiting, fever, headache, skin rash, neck pain	Thoracic			1059 (95%)	M	Acyclovir (4 days)	[40]
27	14	Girl	Wild	paresthesias, hyperesthesia and sensory changes, fever, headache, meningeal signs	No rash	59	49	434 (60%)	M/E	Acyclovir I.V. (14 days)	[41]
28	14	Boy	Wild	No details	No rash	no info	no info	no info	M	no info	[24]
29	15	Girl	Wild	No details	Yes, no details	no info	no info	no info	M	no info	[24]
30	15	Girl	Wild	No details	Yes, no details	no info	no info	no info	M	no info	[24]
31	16	Girl	Wild	No details	No rash	no info	no info	no info	M	no info	[24]
32	16	Girl	Wild	No details	No rash	no info	no info	no info	M	no info	[24]
33	9	Boy	Wild	headache, vomiting, photophobia, billateral papilledema	No rash	31	60	276	P/C	No treatment	[42]
34	15	Boy	Wild	headache, neusea, photophobia, billateral papilledema	No rash			0	P/C	No treatment	[42]
35	11	Boy	Wild	headache, visual obsxuration, vomiting, billateral papilledema	No rash	47	43	7	P/C	No treatment	[42]
36	14	Boy	Wild	fever, headache, slowness, drowsiness, vomiting, skin rash, meningeal signs	Cervical	95	48	1400	M	Acyclovir P.O. (2 days) and acyclovir I.V. (10 days)	[43]
37	17	Girl	Wild	fever, drowsiness, slowness, headache, diplopia	No rash	70		220	M/E	Acyclovir I.V. (14 days) and acyclovir P.O. (7 days)	[44]
38	15	Girl	Wild	headache, new-onset diplopia, blurring of vision, nausea, vomiting	No rash	62	40	537 (96%)	M	Acyclovir I.V. (14 days)	[45]
39	12	Boy	Wild	skin rash, headache, hyperemia of the left bulbar conjunctiva	Trigeminal	24	63	33 (100%)	M	Acyclovir I.V. (14 days)	[16]
40	12	Boy	Wild	ocular pain, skin rash, headache	Trigeminal	33	57	36 (97%)	M	Acyclovir IV (14 days) and famciclovir PO (no duration details)	[16]
41	7	Boy	Wild	headache, vomiting, photophobia, somnolence, skin rash	Thoracic	90	41	480 (97%)	M	Acyclovir I.V. (14 days)	[46]
42	11	Girl	Wild	headache, vomiting, skin rash, meningeal signs	Thoracic	36	47	429	M	Acyclovir I.V. (3 days) and valacyclovir P.O. (10 days)	[47]
43	15	Girl	Wild	fever, headache, nocturnal awakening, photophobia, pain and itch overvaginal and anal region, meningeal signs	Sacral	79	52	34	M	Acyclovir (10 days)	[15]
44	12	Girl	Wild	headache, fever, vomiting, photophobia, cough, rhinitis, psychotic symptoms, altered mental status	No rash	72	52	484 (79%)	E	Acyclovir I.V. (14 days) and acyclovir P.O. (14 days)	[48]
45	13	Boy	Wild	abdominal pain, vomiting, fever, headache, photophobia	No rash	150	39	445 (96%)	M	Acyclovir I.V. (21 days)	[49]
46	14	Boy	Wild	headache, photophobia, vomiting, fever	No rash	130		573 (99%)	M	Acyclovir I.V. (21 days)	[49]
47	16	Boy	Wild	headache, fever, vomiting, fatigue, sore thorat, coryza, drowsiness, photophobia, meningeal signs	Thoracic	90	45	184 (100%)	M	Acyclovir I.V. (14 days)	[49]
48	14	Boy	Wild	headache, vomiting, fever, meningeal signs	No rash	27	77	60 (80%)	M	Acyclovir I.V. (10 days)	[50]
49	14	Boy	Wild	right-sided facial drop, dysphasia, confusion, agitiation, coryza, nasal congestion, left-sided headache, vomiting	No rash				M/E	Acyclovir I.V. (no details)	[17]
50	16	Girl	Wild	headache, rash, vomiting, meningeal signs	Thoracic	221	53	660 (98%)	M	Acyclovir I.V. (21 days)	Our patient no. 1
51	17	Boy	Wild	headache, vomiting, meningeal signs	No rash	170	51	522 (93%)	M	Acyclovir I.V. (21 days)	Our patient no. 2
52	13	Girl	Wild	headache, rash, vomiting, altered mental status	Cervical	119	44	930 (98%)	M/E	Acyclovir I.V. (16 days)	Our patient no. 3
53	13	Girl	Wild	headache, vomiting, skin rash, vomiting	Trigeminal	38	62	23 (98%)	M	Acyclovir I.V. (21 days)	Our patient no. 4
54	16	Boy	Wild	faver, headache, skin rash, vomiting	Thoracic	50	61	408 (83%)	M	Acyclovir I.V (21 days)	Our patient no. 5
55	15	Girl	Wild	fever, headache, rash, vomiting, photophobia	Cervical	49	47	537 (94%)	M	Acyclovir I.V (19 days)	Our patient no. 6

The table includes all reported cases from the literature (cases 1 to 49), along with 6 cases of children hospitalized in Bialystok, Poland (cases 50 to 55). The virus type was classified as “wild” if there was no history of vaccination or immunization. If the vaccine strain was detected in the cerebrospinal fluid (CSF) sample, the child was categorized as having a “confirmed vaccine strain.” If there was no history of chickenpox or varicella vaccination, the child was categorized as having a “presumed vaccine strain”.

### 3.2. Clinical Presentation

**Figure 2:**
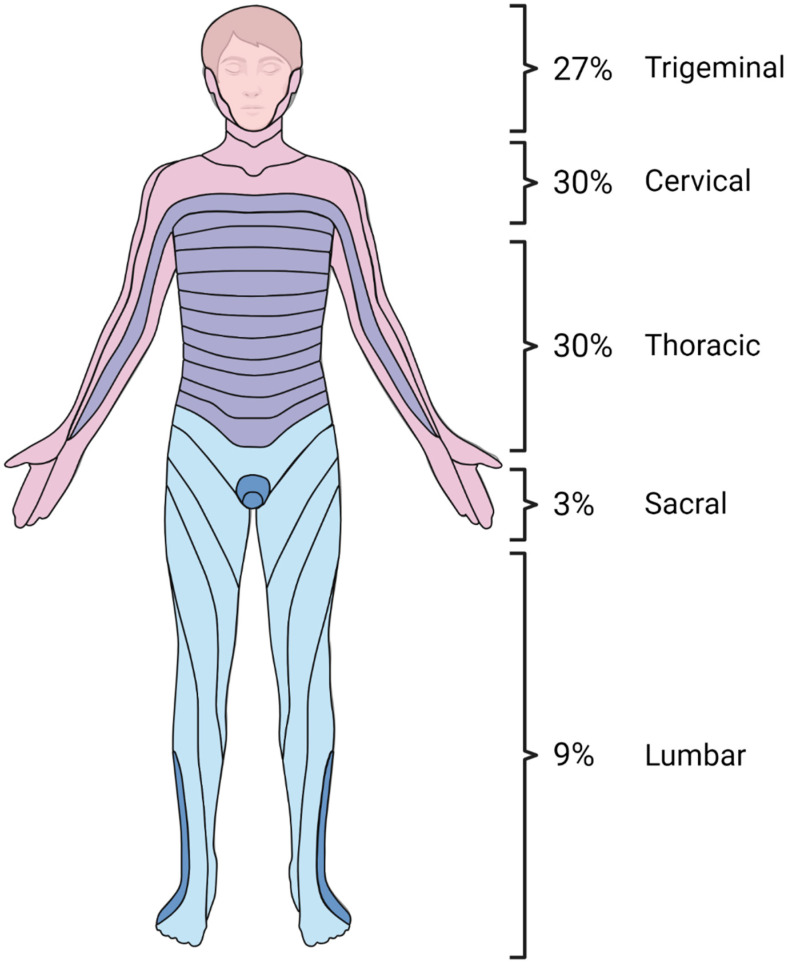
Dermatomal distribution of skin lesions in herpes zoster neuroinfections in immunocompetent children. Created using www.BioRender.com.

There were no significant differences in clinical presentation between the study groups (Table 2). A typical herpes zoster (HZ) rash was observed in only 32 out of 55 children (58%). Information about rash location was available for 30 out of 32 children (94%). The most common rash locations were the cervical and thoracic regions (both 30%), followed by the trigeminal region (27%), lumbar dermatome (9%), and sacral dermatome (3%) (Figure 2). In two children, the rash was disseminated. One case involved a 12-year-old boy whose rash began in the lumbar area and gradually spread to his trunk and scalp. The second case was a 5-year-old girl whose rash started on her face and spread to her trunk. CNS Imaging and EEG.

Information about CNS imaging was provided in 31 patients. Twenty-six (84%) of the patients had normal computed tomography (CT) or magnetic resonance imaging (MRI). Only four (16%) had some intracranial changes in MRI—two patients had meningeal enhancement suggestive of meningitis, one had bilateral multifocal changes in white and grey matter predominantly on the fronto-parietal cortex, and one had hyperintense lesions without enhancement on the right side. One patient had changes consistent with sinusitis in MRI and CT. Four patients had changes in EEG. Two of them had abnormalities consistent with encephalitis, one had right-hand sided slow waves, and one had severe alteration of cortical electrogenesis with exacerbation of diffuse slow cortical activity, with fronto-temporal predominance.

### 3.3. Treatment and Outcome

Of the 55 children, 41 (75%) received antiviral treatment. Approximately 34 children (62%) were treated with acyclovir alone (monotherapy), with 30 of them receiving intravenous acyclovir exclusively and 4 receiving intravenous acyclovir followed by an oral formulation. Six children (11%) were treated with both intravenous acyclovir and oral valacyclovir, while one child (2%) received intravenous acyclovir and oral famciclovir. The total duration of antiviral treatment varied between 4 days and 28 days (median 14 days). Five children did not receive any antiviral treatment. Information about the treatment could not be obtained in nine patients.

The outcome was favorable for most patients—80% of them were discharged without any sequelae. In seven cases, there was no information about the outcome. Four patients had complications from the disease. Two had persistent fatigue, one mild hemiparesis, and one had sensorineural hearing loss. 

Four of five patients who did not receive any antiviral treatment also recovered without any complications. Only one untreated patient had mild fatigue after discharge.

## 4. Discussion

The vast majority of HZ cases with CNS involvement are caused by the reactivation of the wild virus. Although the Varicella-Zoster Virus vaccine demonstrates efficacy in preventing chickenpox and shingles, it is crucial to acknowledge that the vaccine strain, like the wild VZV, can also reactivate, leading to neuroinfections. Even the history of VZV immunization in individuals who have not experienced chickenpox does not rule out VZV meningitis.

Generally, VZV is a rare causative agent of aseptic meningitis in the pediatric population. A comprehensive study conducted in our clinic revealed that only 3% of neuroinfection cases were attributed to VZV [51]. This finding is consistent with similar investigations showing that the frequency of VZV detection in CSF fluctuates in around 2% of pediatric neuroinfection cases [52]. Further research on the epidemiology of VZV reactivations in the pediatric population also shows that CNS involvement is rare, accounting for approximately 5 to 10% of severe Herpes Zoster (HZ) cases [53,54]. Nevertheless, an alternate perspective emerges when considering the adult population, in whom reports indicate VZV reactivation as a leading causative agent for meningitis and encephalitis [24,55]. In light of these considerations, we suggest including VZV in the infectious workup, particularly after eliminating more commonly implicated agents in CNS infections. This recommendation gains particular relevance in cases where there is a documented history of childhood chickenpox.

No discernible differences were observed in the clinical presentation, laboratory features, or outcomes between the cases of HZ induced by the wild and vaccine strains of VZV. However, there was a noticeable difference in the age at which the children experienced virus reactivation. Children experiencing VZV neuroinfection subsequent to VZV vaccination exhibited a younger median age in comparison to those who encountered primary chickenpox (median age of 9 years for the vaccine strain versus 14 years for the wild virus) [8,56]. According to The Summary of Product Characteristics (SmPC), the VZV vaccine is indicated for individuals aged 12 months and older. This stands in contrast to the median age of primary infection in unvaccinated individuals, which typically occurs at 3 years or later [57,58]. The similarity in the interval periods for wild VZV and vOKA (vaccine strain) reactivation implies that the variance in the age at reactivation onset may be attributable to the distinct age at initial contact with the virus. Thus, we presume that the immune mechanisms governing the reactivation of the wild and vaccine strains do not exhibit substantive differences.

No difference in the frequency of zoster rash was observed between the two types of VZV. We emphasize that some children may not manifest the typical vesicular rash characteristic of the disease. Notably, our meta-analysis revealed that 42% of VZV neuroinfection cases transpired without an accompanying rash. The phenomenon of Zoster sine herpete, denoting VZV reactivation without evident skin lesions, has been documented in the existing literature [24,59,60]. Particularly CNS reactivations may tend to present as meningitis or encephalitis without concomitant skin involvement. Furthermore, the dermatomal distribution does not influence the occurrence of VZV reactivation in the CNS. However, in one study, researchers noted that a skin rash with craniocervical distribution was associated with a heightened risk of aseptic meningitis [61]. It is crucial to consider VZV as a potential etiological factor in cases of aseptic meningitis where no definitive causative agent has been established, even in the absence of an overt rash. The lack of a typical HZ rash should not preclude the consideration of VZV reactivation as a plausible causative factor.

In our study, we wanted to highlight that HZ neuroinfection is not exclusive to individuals with recognized risk factors for VZV reactivation in the CNS. While our findings are based on a small sample size and few case reports, they align with other research showing that VZV reactivation can occur in children who appear healthy. The most well-established risk factors are compromised immune status and the use of immunosuppressive medications. Consequently, numerous studies detailing VZV reactivations with CNS involvement predominantly feature patients with compromised immunity [3,28]. However, it is noteworthy that none of the patients included in our meta-analysis exhibited signs of immunocompromise. This finding aligns with the observations of other researchers who have similarly documented that a majority of HZ and VZV reactivations in the CNS occur in previously healthy individuals [62,63]. Some studies even indicate that children without underlying immunodeficiencies are emerging as the predominant demographic among patients experiencing both HZ and CNS infections [62,63,64]. We must remember that every healthy child may develop HZ meningitis. 

The last important aspect is the management of VZV reactivation. The diagnostic approach recommended by the International Herpes Management Forum suggests performing a Real-Time PCR analysis of the CSF when VZV infection is suspected. This test is marked by a high sensitivity of 90% [65]. Alternatively, the detection of VZV-specific IgG in the CSF is proposed as a substitute test [65,66]. Some authors even advocate for this test as a potentially more sensitive indicator of VZV infection compared to the detection of VZV DNA in the CSF [67,68]. Based solely on the CSF parameters, the distinction between wild and vaccine strain infections is not feasible. The definitive confirmation of vaccine virus reactivation necessitates the performance of strain genotyping.

Treatment of VZV meningitis remains a significant challenge. To date, the standards for treating VZV meningitis have not been established. Current clinical practice guidelines published by the Infectious Diseases Society of America recommend the administration of I.V. acyclovir at a dosage of 10–15 mg/kg every 8 h for 10–14 days in the treatment of VZV CNS infection [69]. Our study data indicate a median treatment duration of 14 days. Nevertheless, there is a lack of empirical evidence demonstrating a discernible alteration in the disease course with the use of acyclovir in VZV reactivation within the CNS. Notably, in all cases under investigation, symptoms were alleviated and outcomes were favorable irrespective of whether the patient received a 14-day course of treatment or no treatment at all. The question of whether VZV reactivation in the CSF is a self-limiting condition that does not necessitate extended treatment remains unresolved. The matter of treatment in VZV neuroinfections continues to be a subject of ongoing research, and until new therapeutic standards are established, acyclovir remains the drug of choice for such conditions.

The limitations of our study include the reliance on published case reports and case series, which may introduce selection bias and limit the generalizability of our findings. Additionally, the retrospective nature of the study and the small sample size of our cohort may affect the robustness of our conclusions. Furthermore, the variability in reporting and diagnostic criteria among the included studies could introduce heterogeneity in the data analysis. Moreover, the lack of long-term follow-up data in some cases may hinder our ability to assess the full spectrum of clinical outcomes associated with VZV reactivation in the CNS. Additionally, the possibilities of underreporting and misdiagnosing VZV-related CNS infections in the literature and in our hospital records cannot be completely ruled out. Finally, while efforts were made to ensure the accuracy of the genotype analysis for confirming vaccine strain reactivation, the potential for technical limitations and errors in this process cannot be entirely eliminated.

In conclusion, our study highlights the significance of Varicella-Zoster Virus (VZV) reactivation as a cause of central nervous system (CNS) infections in immunocompetent children. We observed a considerable proportion of cases involving both wild-type and vaccine strain reactivation, underscoring the importance of considering VZV in the diagnostic workup of CNS infections regardless of vaccination status. Clinical presentation varied, with a typical herpes zoster rash observed in just over half of the cases, often localized in cervical and thoracic dermatomes. Despite the heterogeneity in the CNS imaging findings and EEG abnormalities, the majority of patients received antiviral treatment, resulting in favorable outcomes for most, with few experiencing complications. These findings emphasize the need for heightened awareness among health care professionals regarding the potential for VZV reactivation in immunocompetent children presenting with CNS symptoms, facilitating timely diagnosis and appropriate management to optimize patient outcomes.

## Figures and Tables

**Table 2:** Summary characteristics of the study groups.

	Wild-Type Virus (n = 37)	Vaccine-Strain Virus (n = 18)	*p*
Sex, girls, n (%)	16/36 (43%)	6/18 (33%)	0.433
Age, years, median (min-max)	14 (2–17)	10.5 (2–18)	0.292
Interval, years, median (min-max) *	10 (1.7–12.5)	8 (1.0–14.0)	0.802
Fever, n (%)	14/29 (48%)	13/17 (76%)	0.061
Herpes zoster, n (%)	19/37 (51%)	13/18 (72%)	0.184
Headache, n (%)	29/29 (100%)	14/17 (82%)	0.019
Nausea and vomiting, n (%)	23/28 (82%)	9/17 (53%)	0.036
Altered mental status, n (%)	9/29 (31%)	2/16 (13%)	0.166
Focal neurological signs, n (%)	8/29 (28%)	1/17 (6%)	0.073
Meningeal signs, n (%)	9/29 (31%)	9/17 (53%)	0.142
Photophobia, n (%)	12/28 (43%)	5/17 (29%)	0.367
EEG abnormalities	1/4 (25%)	0/1 (0%)	0.576
MRI/CT abnormalities	4/24 (17%)	1/7 (14%)	0.880
CSF Protein, mg/dLmedian (min-max)	71 (24–221) (n = 24)	62 (10–137) (n = 12)	0.920
CSF cells/µLmedian (min-max)	429 (0–1400) (n = 27)	243 (0–775) (n = 14)	0.847
CSF lymphocytes, %median (min-max)	96 (60–100%) (n = 19)	88 (71–97) (n = 11)	0.106

* In the wild-type virus group, the interval describes the number of years between primary infection with the VZV and reactivation; in the vaccine-strain group, the interval describes the number of years between the first dose of vaccine and reactivation; denominators lower than the group size indicate incomplete data.

## Data Availability

The data presented in this study are available on request from the corresponding author.

## Supplementary Materials

The following supporting information can be downloaded at https://www.mdpi.com/article/10.3390/vaccines12091086/s1, File S1: Preferred Reporting Items for Systematic reviews and Meta-Analyses extension for Scoping Reviews (PRISMA-ScR) Checklist. Reference [14] is cited in the supplementary materials.
